# Long noncoding RNA RP11-241J12.3 targeting pyruvate carboxylase promotes hepatocellular carcinoma aggressiveness by disrupting pyruvate metabolism and the DNA mismatch repair system

**DOI:** 10.1186/s43556-021-00065-w

**Published:** 2022-02-05

**Authors:** Liuliu Cheng, Shichuan Hu, Jinhu Ma, Yongheng Shu, Yanwei Chen, Bin Zhang, Zhongbing Qi, Yunmeng Wang, Yan Zhang, Yuwei Zhang, Ping Cheng

**Affiliations:** 1 State Key Laboratory of Biotherapy and Cancer Center/Collaborative Innovation Center for Biotherapy, West China Hospital, Sichuan University, 17 People’s South Road, Chengdu, 610041 Sichuan PR China; 2 Department of Thoracic Oncology, Cancer Center and State Key Laboratory of Biotherapy, West China Hospital, Sichuan University, Chengdu, 610041 Sichuan PR China; 3 Division of Endocrinology and Metabolism, State Key Laboratory of Biotherapy, West China Hospital, Sichuan University, Chengdu, 610041 Sichuan PR China

**Keywords:** Hepatocellular carcinoma, HBx, LncRNAs, RP11-241J12.3, DNA MMR, Pyruvate metabolism

## Abstract

Accumulating evidence indicates that hepatitis B virus X protein (HBx) plays a key role in HBV-related hepatocellular carcinoma (HCC) aggressiveness; however, the underlying mechanisms are not entirely clear. Long non-coding RNAs (lncRNAs), which participate in the regulation of diverse biological processes, may be critical for the function of HBx. Our research indicated that HBx induced changes in the expression of numerous lncRNAs and implicated the novel lncRNA RP11-241J12.3 in HBx-mediated HCC aggressiveness. Although RP11-241J12.3 expression was downregulated in transient HBx-expressing HCC cells (similar to the early stage of HBV infection), its oncogenic properties remained. The results showed that RP11-241J12.3 not only accelerated DNA synthesis and upregulated the expression of pyruvate carboxylase (PC) and MSH3, which is a key protein in pyruvate metabolism and DNA mismatch repair (MMR), but also promoted tumor growth *in vitro* and *in vivo*, thus promoting HCC aggressiveness. More importantly, we revealed that RP11-241J12.3 may interact with PC and identified its location in the cytoplasm close to the nucleus using fluorescence in situ hybridization (FISH). We also observed RP11-241J12.3 expression was upregulated in HCC tissues compared with the paracarcinomatous tissues. Furthermore, RP11-241J12.3 expression levels showed a close relationship with clinical stage and tumor size and that low RP11-241J12.3 expression was significantly correlated with longer HCC patient survival. These results further our understanding of the lncRNAs regulated by HBx in HCC, and provide evidence that dysregulation of RP11-241J12.3 contributes to HCC aggressiveness.

**Supplementary Information:**

The online version contains supplementary material available at 10.1186/s43556-021-00065-w.

## Introduction

As one of the most common cancers with increasing morbidity, hepatocellular carcinoma (HCC) is the sixth most common malignancy and the second most frequent cause of cancer death in the word [1]. More than 250,000 cases of new HCC and about 600,000 people’s lives are threatened by HCC. Chronic hepatitis B virus (HBV) infection is the most important risk factor for hepatocarcinogenesis [2]. Recent reports claim that at least 250 million people worldwide are chronically infected with HBV [3]. Hepatitis B virus X protein (HBx) has been verified to act as a multifunctional regulator of HCC through its influence on cell cycle progression, apoptosis, and transcriptional regulation [4–6]. Our previous study revealed that HBx induces DNA damage and interferes in glucose, lipid and nucleic acid metabolism [7]. However, the molecular mechanism of HBx mediated hepatocarcinogenesis remains largely unclear, especially the mechanism by which HBx causes metabolic disturbance and DNA damage.

Long non-coding RNAs (lncRNAs) are a subgroup of non-coding RNAs that are defined as longer than 200 nucleotides with low or no protein-coding capacity. LncRNAs play significant roles in biological processes including cell cycle progression and apoptosis, signaling pathways, tumor progression and metastasis [8]. Recent studies also indicated that lncRNAs represent a novel therapeutic target based on their contribution to chromatin architecture, regulating post-transcriptional RNA processing, and DNA methylation [9]. Some lncRNAs have been characterized as having tumor suppressor or oncogenic properties related to HBx in HCC. HULC, a lncRNA expressed at high levels in HCC, was shown to be upregulated by HBx and accelerate the progress of HCC by inhibiting PTEN via autophagy cooperation to miR15a [10]. Another lncRNA, Dreh, inhibited themetastasis of HCC by repressing the expression of intermediate filament protein vimentin, which was downregulated by HBx in mouse [11]. Futhermore, the long intervening noncoding RNA-UFC1 (lincRNA-UFC1), which is upregulated in HCC tissues, promoted cell proliferation and reduces apoptosis [12]. All of these lncRNAs related to HBx have vital functions in the occurrence and development of HCC. Therefore, we hypothesized that lncRNAs might have vital functions in the mechanism of HBx induced metabolic disorder and DNA damage in HCC.

In this study, we used gene microarray and bioinformatics analyses to identify a novel lncRNA RP11-241J12.3 with proto-oncogenic properties and investigated its relationship with HBx-mediated carcinogenesis. We found that HBx led to the abnormal expression of lncRNA RP11-241J12.3. The fuction of lncRNA RP11-241J12.3 had not been reported. Here, we found that lncRNA RP11-241J12.3 can disrupt pyruvate metabolism and damage the DNA MMR system through inducing upregulation of pyruvate carboxylase (PC) and MSH3 expression, finally resulting in DNA damage that promoted HCC aggressiveness. Furthermore, the high lncRNA RP11-241J12.3 expression in HBV-associated HCC tissues was close relationship with shorter HCC patient survival in clinical. Thus, lncRNA RP11-241J12.3 could be used as a marker to judge the prognosis of HCC and a potential therapeutic target for HCC.

## Results

### HBx regulated the expression of diverse lncRNAs

**Fig. 1:**
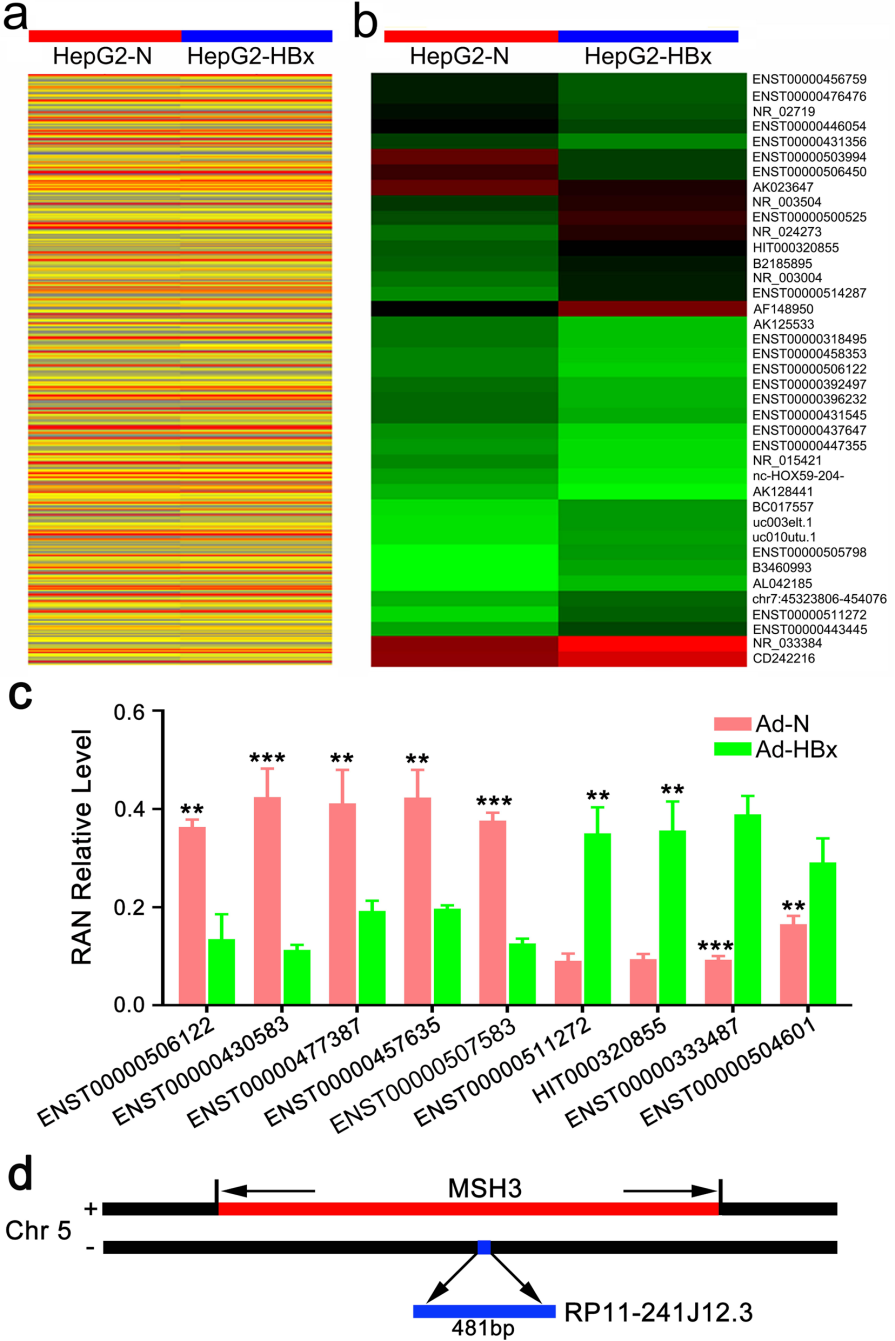
HBx regulated the expression of diverse lncRNAs in HepG2 cells. **a** Expression of diverse lncRNAs changed by HBx. **b** Significantly upregulated (cutoff ≥3-fold) and significantly downregulated (cutoff ≤3-fold) lncRNAs. Red or Green color on the heat map indicates a decrease or an increase in the lncRNA level and the color intensity corresponds to the relative signal levels on a logarithmic scale. **c** Nine random lncRNAs were verified by qRT-PCR. Data are normalized to *GAPDH* and represent the mean ± SD of three replicate experiments. ENST00000506122 is designated lncRNA RP11-241J12.3. **d** LncRNA RP11-241J12.3 is close to the primary transcript of MSH3 in the genome (magnification 1:200)

LncRNAs that function in cellular processes can be identified by transcriptome, microarray and bioinformatical analyses. To examine a series of lncRNAs related to HBx functional processes, we identified genome-wide lncRNA expression in HepG2 HCC cells Ad-HBx– or Ad-N–infected HepG2 HCC cells using microarrays. After infection and culture for 48 h, cells were harvested and the lncRNA expression profiles were detected using human lncRNA microarrays (probes for 33,000 lncRNAs) using fold-change in expression >2 as a threshold. Statistical analysis revealed that a total of 632 lncRNAs were upregulated, while 448 lncRNAs were downregulated (Fig. 1a). We analyzed the data for all of the changed lncRNAs and selected some of the upregulated (cutoff ≥3-fold) and downregulated (cutoff ≤3-fold) lncRNAs to further analysis (Fig. 1b). To validate the reliability of the lncRNA microarray results, we also randomly selected nine lncRNAs to validate the changes in expression by qRT-PCR (Fig. 1c). The detection results of the two methods were consistent. Thus, our results revealed that HBx caused widespread changes in the expression of abundant lncRNAs.

Our previous studies indicated that HBx induced G2/M arrest, inhibited DNA synthesis, and cause DNA damage and abnormalities of nucleic acid metabolism; therefore, we hypothesized that HBx acts during prophase to suppress HCC proliferation, although this opinion is controversial [7, 13]. To investigate the relationship between the aberrantly expressed lncRNAs and the function of HBx, we analyzed the upstream and downstream lncRNAs in the genome and identified lncRNA RP11-241J12.3 as a novel lncRNA related to the function of HBx. RP11-241J12.3 was significantly downregulated in Ad-HBx–infected HepG2 cells compared with the levels detected in Ad-N–infected cells. RP11-241J12.3 (NONCODE ID: NONHSAG040861.2, UCSC ID: ENST000000506122.1, chr5:8074339-80746819) was shown to be a 481-bp antisense strand lncRNA, and the sense strand at the same site contained the primary transcript of MSH3, which played a crucial role as a key multifunctional protein in DNA MMR (Fig. 1d). Our previous study reported that HBx downregulate the expression of MutL homolog 1 (MLH1) in the MMR system [7]; therefore, we hypothesized that RP11-241J12.3 regulates the MMR system, and plays a vital role in HBx-related HCC.

### HBx downregulated the expression of proteins in the pyruvate metabolism and MMR systems

**Fig. 2:**
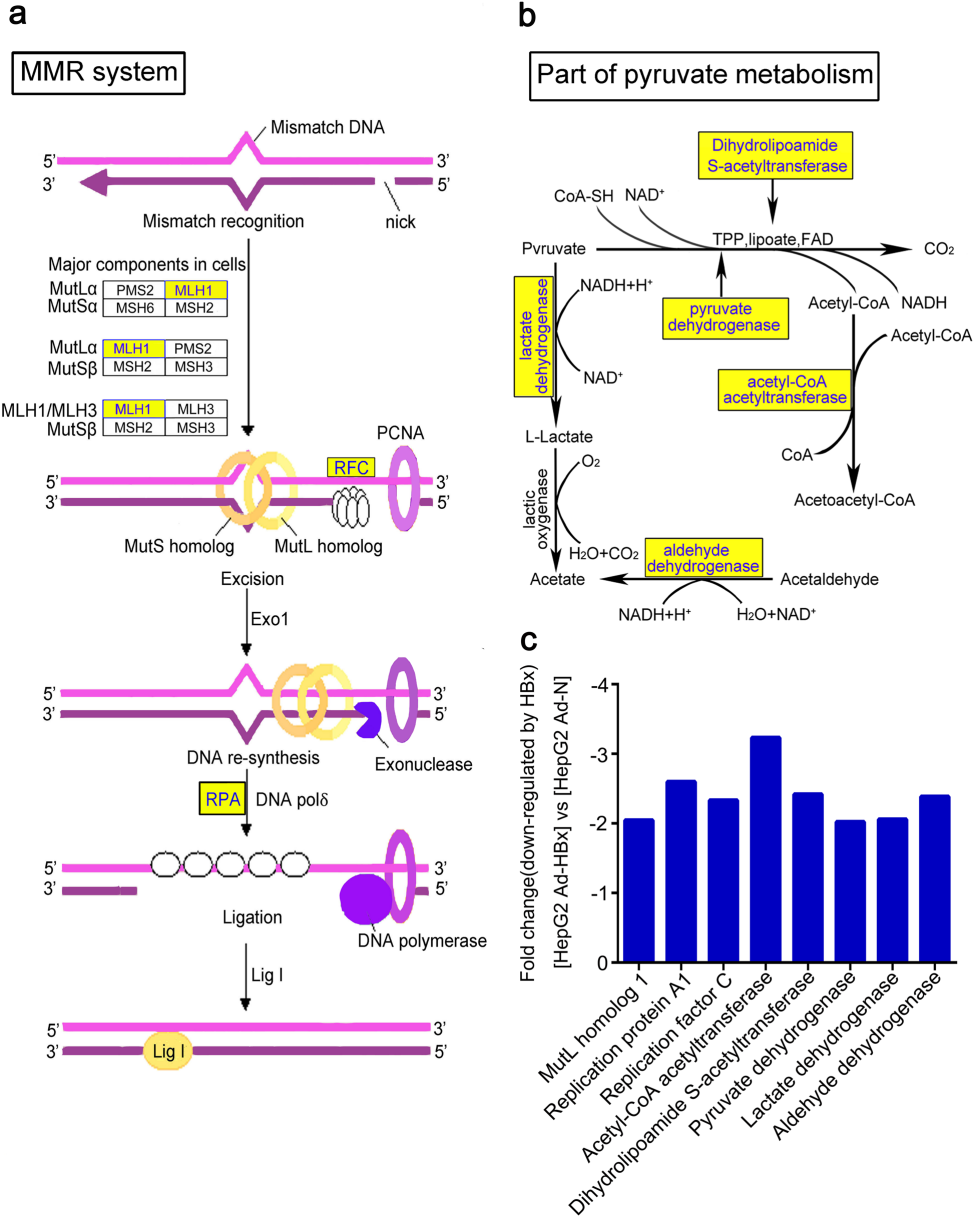
HBx downregulated the expression of key proteins in the DNA MMR and pyruvate metabolism systems. **a-b** Downregulated proteins (blue) played vital roles in the DNA MMR and pyruvate metabolism systems. MLH1, replication factor C (RFC) and replication protein A1(RPA) in the DNA MMR system, and dihydrolipoamide S-acetyltransferase, lactate dehydrogenase, pyruvate dehydrogenase, acetyl-CoA acetyltransferase and aldehyde dehydrogenase in the pyruvate metabolism system were downregulated. **c** Fold-change downregulation of HBx in HepG2 cells. All proteins was investigated by pathway analysis mapping genes to KEGG pathways

According to the previous report that HBx changed the expression of some mRNAs [7], we further analyzed the expression of mRNAs in Ad-HBx– or Ad-N–infected HepG2 cells using microarrays. In the MMR system, MLH1, RFC and RPA were downregulated (Fig. 2a). In the DNA MMR system, which is indispensable for cell to maintain the genomic stability, MutS encodes MSH2, MSH3 and MSH6, and MutL encodes MLH1, MLH3, PMS1 and PMS2 in humans [14]. It has previously been reported that HBx downregulates the expression of MLH1 in the MMR system [7]. Downregulation of these proteins would result in serious impairment of the DNA MMR system, resulting in genomic DNA damage.

Our results also showed that HBx downregulated five important proteins (dihydrolipoamide S-acetyltransferase, lactate dehydrogenase, pyruvate dehydrogenase, acetyl-CoA acetyltransferase, and aldehyde dehydrogenase) in the pyruvate metabolism system (Fig. 2b); these results were verified by qPCR (Fig. 2c). Pyruvate is the end-product of the glycolytic pathway that produces energy and is vital for carbohydrate, lipid and protein production. Thus, pyruvate metabolism is critical for maintenance of the balance of nutrients. Downregulation of enzymes in the pyruvate metabolism pathway account for disruption of pyruvate metabolism, especially aerobic oxidation, which is required for energy production. Previous studies indicated that pyruvate reduced DNA damage in HCC cells [15]. On the basis of our lncRNA array analyses, we hypothesized that lncRNA RP11-241J12.3 plays an extremely important role in pyruvate metabolism or the DNA MMR system.

### Upregulation of RP11-241J12.3 promoted HCC cell proliferation, migration and invasion

**Fig. 3:**
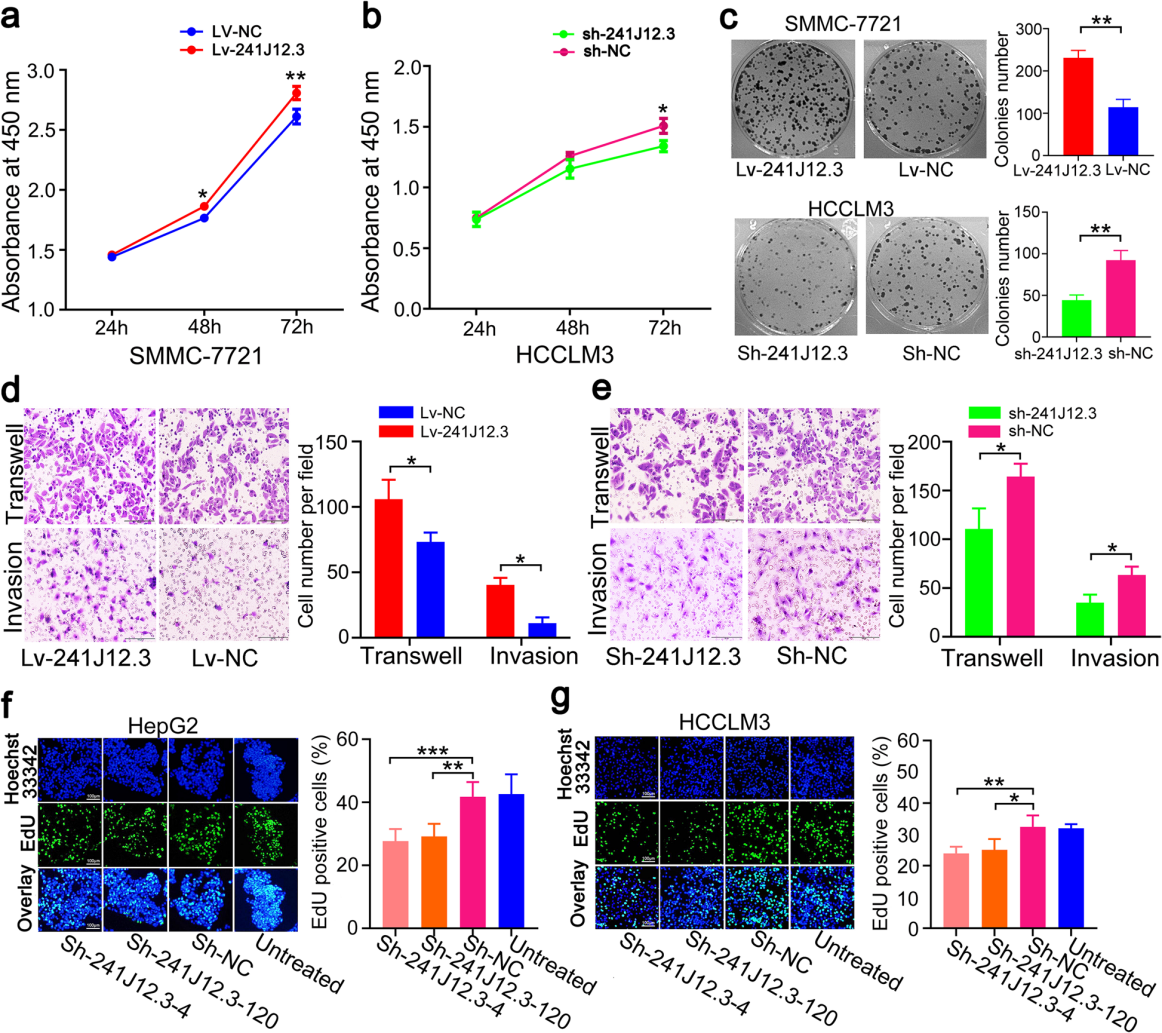
Upregulation of RP11-241J12.3 stimulated HCC cells colony-formation migration and invasion, and accelerated DNA synthesis. **a** Cell colony formation assays showing that RP11-241J12.3 overexpression increased SMMC-7721 cell colonies; the opposite was observed after RP11-241J12.3 knockdown. Specific analysis is shown on the right. **b-c** After separate overexpression of RP11-241J12.3 in SMMC-7721 cells or silencing it in HCCLM3 cells, the cell viability was assessed by cell proliferation assay. **d-e** After separate overexpression or silencing of RP11-241J12.3, migration of SMMC-7721 cells was evaluated in Transwell assays. Quantification of the cells is shown on the right. **f-g** HepG2 and HCCLM3 cells transfected with ShRNAs (Sh-241J12.3-4 or 120, Sh-NC, untreated) were seeded on 96-well plates and the effect of RP11-241J12.3 knockdown on cell proliferation was evaluated using EdU incorporation assay at 72 h post-transfection. Quantification of EdU-positive nuclei is shown on the right. Data represent the mean ± SD

As a previously unexplored lncRNA, we investigated the biological functions of RP11-241J12.3. First, we explored the ability of RP11-241J12.3 to influence HCC cell biological phenotypes, including cell proliferation, migration and invasion. To detected the ability of RP11-241J12.3 to promote cell proliferation, we used CCK-8 assays to indicate that RP11-241J12.3 overexpression enhanced HCCLM3 cell proliferation, whereas knockdown of endogenous RP11-241J12.3 expression inhibited the SMMC-7721 cell proliferation (Fig. 3a-b). Furthermore, after lentivirus-induced overexpression and shRNA-mediated knockdown of RP11-241J12.3 (Fig. S1a-b), we showed that upregulation of RP11-241J12.3 greatly enhanced the SMMC-7721 cell colony numbers in colony formation assays, while downregulation of RP11-241J12.3 expression had the opposite effect (Fig. 3c). Overall, these findings demonstrated that RP11-241J12.3 enhances HCC cell growth.

We then investigated the influence of RP11-241J12.3 on cell migration and invasion, which was are important functions of HCC cells in liver tumorigenesis. Transwell and invasion assays indicated that the migration and invasion capacity of SMMC-7721 cells was evidently enhanced following RP11-241J12.3 overexpression compared with the negative control, while these functions were inhibited by RP11-241J12.3 knockdown (Fig. 3d-e).

### RP11-241J12.3 accelerated DNA synthesis

Previous studies showed that HBx inhibits DNA synthesis, induces G2/M arrest and apoptosis, and simultaneously reduces the expression of RP11-241J12.3; therefore, we speculated that RP11-241J12.3 also affects DNA synthesis and cell cycle progression during cell proliferation. We tested this hypothesis using EdU incorporation assay and flow cytometric analysis. Cells were transfected with sh-241J12.3-4, sh-241J12.3-120 or sh-NC (pGPU6 /Neo) without GFP-labeling and EdU was incorporated into DNA as an analog of thymidine during DNA synthesis. At 72 h after transfection, the proportions of EdU-labeled cells in the Sh-241J12.3-4 and Sh-241J12.3-120 groups were significantly lower that those in the Sh-NC or untreated groups for both HepG2 and HCCLM3 cells (Fig. 3f-g). Surprisingly, flow cytometric analysis showed that the percentage of cells in G2/M and G0/G1 did not change after RP11-241J12.3 knockdown in HepG2 and HCCLM3 cells. Thus, these findings indicated that RP11-241J12.3 enhanced DNA synthesis, but had no effect on cell cycle progression (Data not show). Overall, we showed that RP11-241J12.3 accelerated DNA synthesis and promoted cell proliferation, migration and invasion *in vitro*.

### RP11-241J12.3 promoted tumor growth *in vivo*

**Fig. 4:**
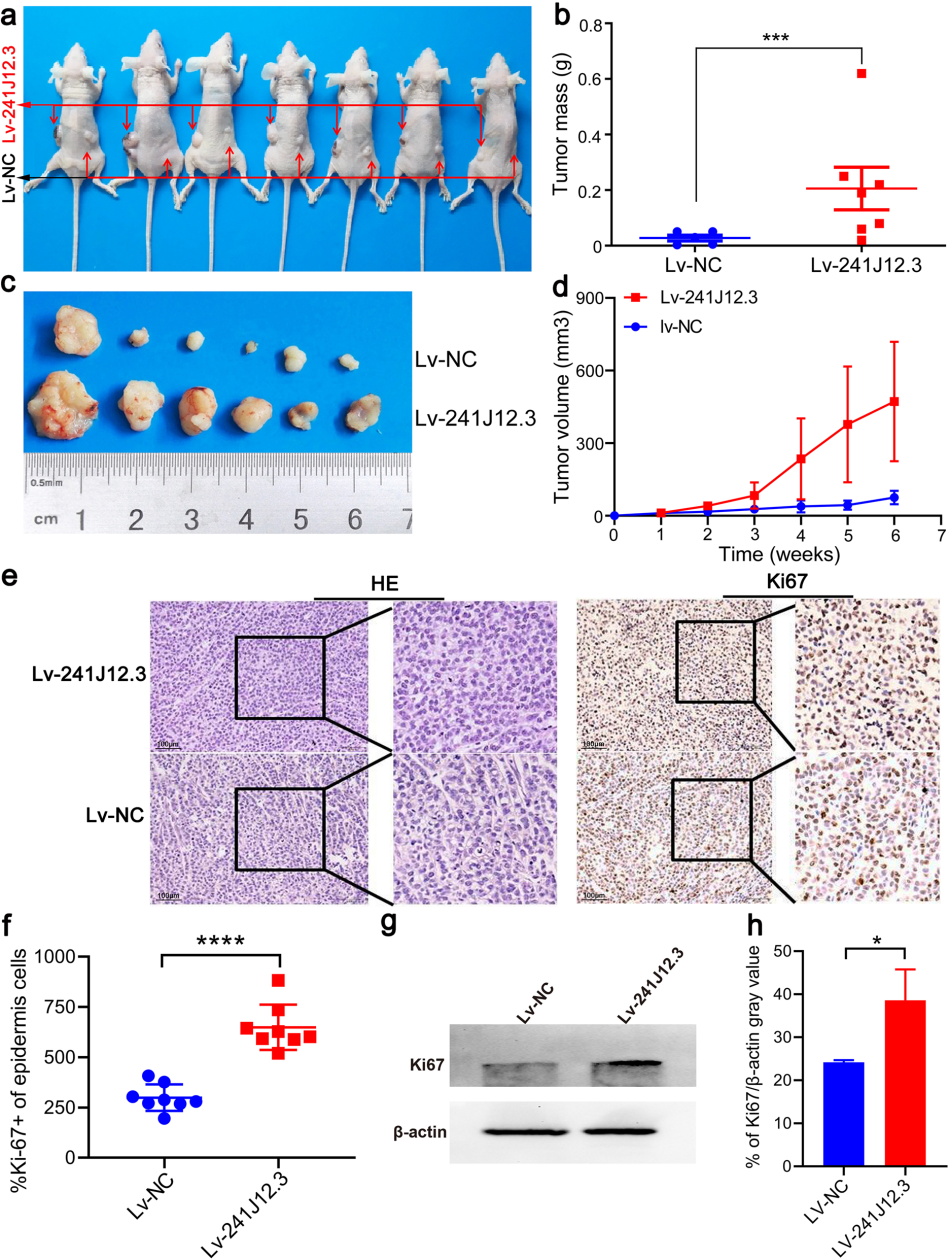
LncRNA RP11-241J12.3 accelerated HCC growth *in vivo*. **a** Images of tumors that developed in nude mice. **b** Tumor growth curves of SMMC-7721 cells transduced with Lv-241J12.3 and Lv-NC in nude mice. Images (**c**) and weights (**d**) of tumor 5 weeks after cell injection. *n* = 7 mices per group. The tumors on both sides subsided in one mouse. **e** Representative images of H&E and IHC staining for tumor tissues (400×). **f** Quantification of IHC staining showing that Ki67 expression was increased in the Lv-241J12.3 group compared to control group. Data were obtained from eight randomly selected fields in four representative slices from each group. **g** Representative images of Western blotting. **h** Quantitative analysis of western blotting. The Ki-67 protein expression in Lv-RP11-241J12.3-derived tumors was significantly higher than that in Lv-control-derived tumors (*n* = 3)

To evaluate the growth promoting effect of RP11-241J12.3 *in vivo*, we transduced SMMC-7721 cells with Lv-RP11-241J12.3 or Lv-NC and generated cell line with stable expression of RP11-241J12.3 cells by puromycin resistance screening. These cells were then injected subcutaneously into nude mice. Abouot 6 weeks after cell inoculation, the cells overexpressing RP11-241J12.3 showed greatly increased tumor growth compared to the that of the cells infected with Lv-NC (Fig. 4a). Furthermore, analysis of tumor volume and mass showed that RP11-241J12.3 overexpression sharply promoted tumor growth *in vivo* (Fig. 4b-d), thus confirming that lncRNA RP11-241J12.3 also promoted tumor growth *in vivo*.

We also performed H&E and Ki-67 immunohistochemistry staining of the xenografted tumors generated following implantation of cells infected with Lv-RP11-241J12.3 and Lv-control. HE staining of the tumors showed that the degree of cell differentiation was lower in Lv-RP11-241J12.3-derived tumors, in which the cell arrangement was disrupted, with irregular heteromorphism, a decreased nucleus/cytoplasm ration, and chromosomes heteropyknosis compared with the control group, in which cell arrangement was orderly. Ki-67 immunohistochemistry staining showed that expression of the Ki-67 proliferation antigen was significantly enhanced in Lv-RP11-241J12.3-derived tumors compared with that in Lv-control-derived tumors (Fig. 4e-f). Morever, Western blot result showed that the Ki-67 protein expression in Lv-RP11-241J12.3-derived tumors was significantly higher than that in Lv-control-derived tumors (Fig. 4g-h). These results indicated a greater level of malignancy of Lv-RP11-241J12.3 tumors compared with that of the control tumors.

### RP11-241J12.3 regulated pyruvate metabolism by targeting PC

**Fig. 5:**
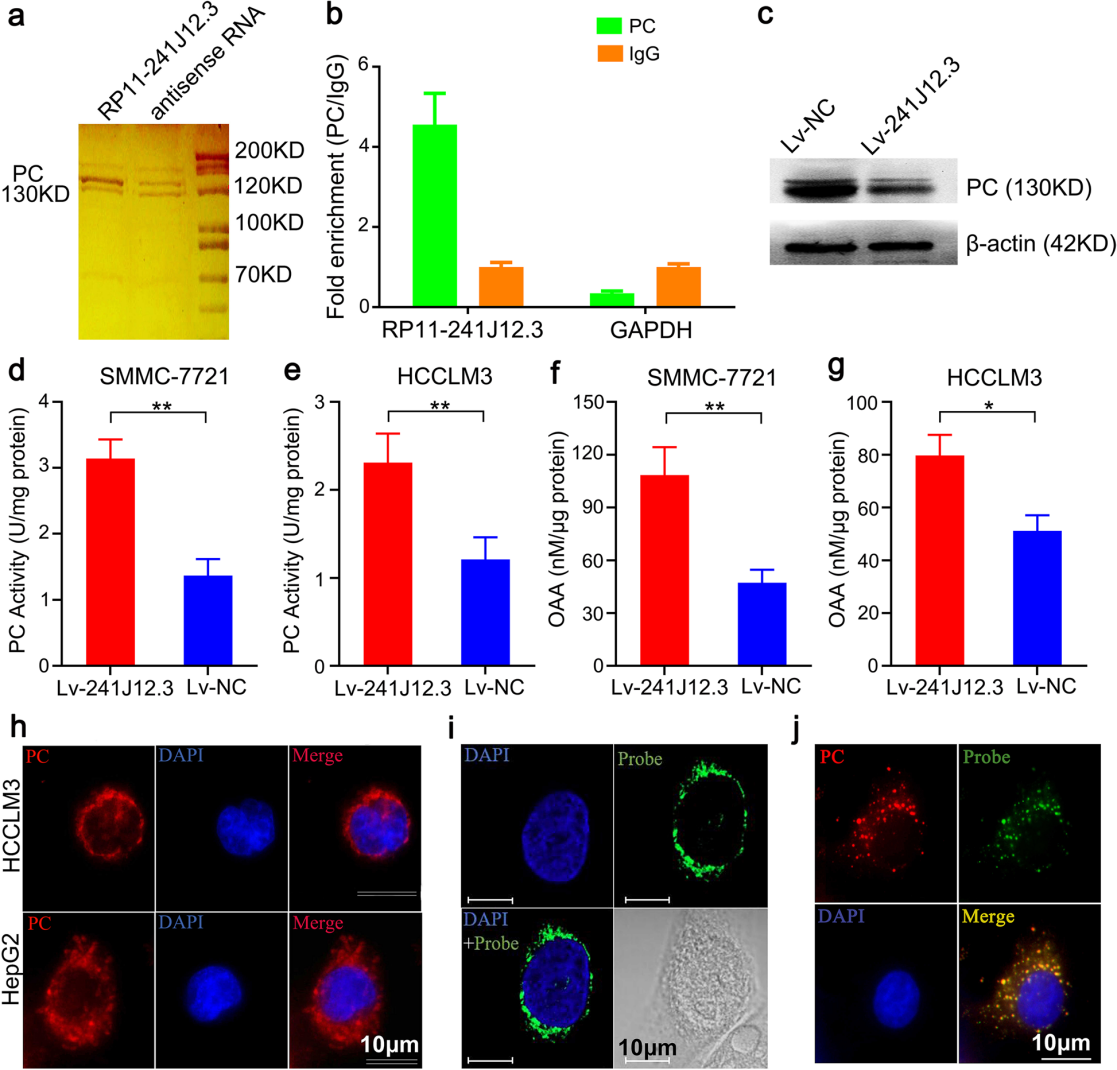
LncRNA RP11-241J12.3 bound directly to PC in the cytoplasm to regulate pyruvate metabolism. **a** RNA pull-down assays with HepG2 cytoplasm extract with specific bands identified by MS. **b** qPCR analysis of the RNAs retrieved by the PC-specific antibody compared with IgG in the RIP assay of HepG2. **c** The protein expression levels of PC were upregulated after RP11-241J12.3 overexpression in HepG2 and SMMC-7721 cells. **d-e** The PC activities were upregulated after RP11-241J12.3 overexpression in SMMC-7721 and HCCLM3 cells. **f-g** The levels of OAA in RP11-241J12.3 overexpressing HCC cells were obviously higher than those of control HCC cells. Data represent the mean ± SD of at least three replicate experiments. **h** Specific location of PC in the cytoplasm of HepG2 and HCCLM3 cells. **i** Location of RP11-241J12.3 in the cytoplasm close to the nucleus in HCCLM3 cells. **j** Colocalization analysis: lncRNA RP11-241J12.3 FISH assay combined with PC immunofluorescence detection

Although we confirmed that RP11-241J12.3 accelerated DNA synthesis and promoted tumor proliferation, the underlying mechanism remained to be elucidated. To identify the target protein of RP11-241J12.3, we performed RNA pull-down assays with HepG2 cell cytoplasmic extracts (Fig. 5a). Using a proteomics strategy and high-resolution LC-MS/MS, we identified 79 proteins in the specific bands visualized by silver staining of gels after gradient electrophoresis (Table 1). Analysis of the relatively accurate MS results implicated PC as the target protein of RP11-241J12.3. PC is a vital protein in pyruvate metabolism located at a crucial crossroads of carbohydrate aerobic metabolism pathways. Importantly, qPCR analysis indicated that lncRNA RP11-241J12.3 was retrieved by the PC-specific antibody but not by the control immunoglobulin G (IgG) in the RNA immunoprecipitation (RIP) assay (Fig. 5b), thus providing further confirmation that PC is the target protein of RP11-241J12.3. Furthermore, RP11-241J12.3 overexpression resulted in upregulation of PC (Fig. 5c), indicating that RP11-241J12.3 participates in pyruvate metabolism through direct regulation of PC expression. It is well known that PC plays a significant role in aerobic oxidation in glycolysis, catalyzes conversion of pyruvate into OAA [16]. Our study showed that the PC activities and the levels of OAA in RP11-241J12.3 overexpressing HCC cells were obviously higher than those of control HCC cells (Fig. 5d-g).

**Table 1:** List of the main proteins in specific bands with RP11-241J12.3 RNA-binding domains identified by LC-MS /MS

Accession	Description	MW [kDa]	#Unique peptides
P42704	Leucine-rich PPR motif-containing protein [LPPRC_HUMAN]	157.8	1
Q9P2E9	Ribosome-binding protein 1 OS = *Homo sapiens* [RRBP1_HUMAN]	152.4	3
O75533	Splicing factor 3B subunit 1 OS = *Homo sapiens* [SF3B1_HUMAN]	145.7	1
Q00341	Vigilin OS = *Homo sapiens* [VIGLN_HUMAN]	141.4	5
**P11498**	**Pyruvate carboxylase, mitochondrial OS =** ***Homo sapiens*** **[PYC_HUMAN]**	**129.6**	**17**
P53396	ATP-citrate synthase OS = *Homo sapiens* [ACLY_HUMAN]	120.8	1
P22314	Ubiquitin-like modifier-activating enzyme 1 OS = *Homo sapiens* [UBA1_HUMAN]	117.8	1
Q14527	Helicase-like transcription factor OS = *Homo sapiens* [HLTF_HUMAN]	113.9	1
Q9HCE1	Putative helicase MOV-10 OS = *Homo sapiens* [MOV10_HUMAN]	113.6	1

Considering the relationship between lncRNA RP11-241J12.3 and PC, we conducted further investigations to characterize RP11-241J12.3 and its function. Immunofluorescence analysis showed that PC was located in the cytoplasm close to the nucleus in hepatoma cells (Fig. 5h). Subsequent FISH studies confirmed that lncRNA RP11-241J12.3 was clearly located in cytoplasm closed to nucleus in HCCLM3 cells (Fig. 5i). Furthermore, PC and RP11-241J12.3 were found to be co-localized in the cells (Fig. 5j). Therefore, we concluded that RP11-241J12.3 participates in PC metabolism as a regulator in the function of HBx. Previous studies showed that some cytoplasmic lncRNAs play a vital role in post-transcriptional regulation, including stabilization of mRNA and translation, or as miRNAs and participating in protein modification as ceRNAs. Thus, it can be speculated that lncRNA RP11-241J12.3 binds PC to regulate the processed mediated by HBx via one of these mechanisms.

As HBx induces metabolic disturbance of glycolysis, and downregulates the expression of lncRNA RP11-241J12.3, which participates in pyruvate metabolism by targeting PC, we primarily speculated that HBx interferes with pyruvate metabolism in the aerobic oxidation that occurs in glycolysis through regulating the expression of RP11-241J12.3 and further promoting HCC aggressiveness.

### RP11-241J12.3 disrupted the DNA mismatch repair system

**Fig. 6:**
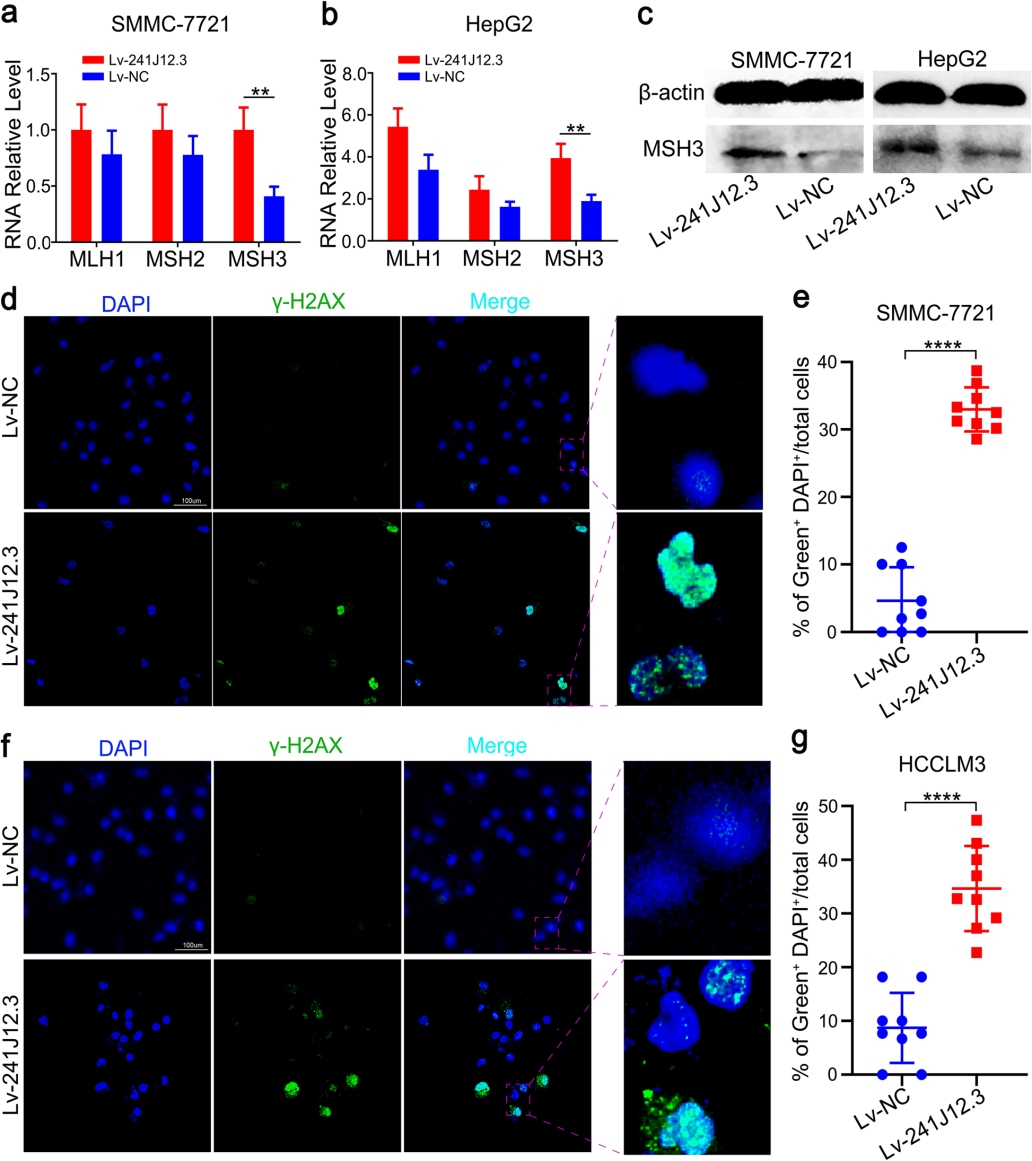
LncRNA RP11-241J12.3 disrupted the MMR system. **a-b** The mRNA levels of MLH1, MSH2 and MSH3 were upregulated after RP11-241J12.3 overexpression in HepG2 and SMMC-7721 cells. lcnRNA RP11-241J12.3 could regulate the expression of PC and MSH3 but not MSH2 or MLH1. **c** Western blot analysis showing upregulation of MSH3 following RP11-241J12.3 overexpression in SMMC-7721 and HepG2 cells. **d** Representative immunofluorescent staining and (**e**) quantification of γ-H2AX in SMMC-7721 cells transduced with Lv-241J12.3 or Lv-NC. **f** Representative immunostaining images and (**g**) statistical analysis of γ-H2AX in HCCLM3 cells transduced with Lv-241J12.3 or Lv-NC. The data represent the mean ± SD of at least three replicate experiments

To clarify the relevance of pyruvate metabolism and the DNA MMR system [15], we also analyzed the influence of RP11-241J12.3 on the DNA MMR system, which is a conserved DNA repair pathway in humans. The fundamental function of MMR is to eliminate the mismatched base-pair caused by deletion and insertion as a result of DNA polymerase errors during DNA synthesis [17]. More importantly, the transcript of the MSH3 gene is similar to RP11-241J12.3, which interacts with MSH2 to participate in DNA MMR systems. Therefore, we also detected the expression of MSH3, HLM1and MSH2 after RP11-241J12.3 overexpression. The results revealed that MSH3 was upregulated by RP11-241J12.3, which had no effect on the expression of MSH2 and HLM1 (Fig. 6a-b). Western blot analysis confirmed that MSH3 was upregulated after RP11-241J12.3 overexpression (Fig. 6c). γ-H2AX was a DNA double-strand break marker. As shown in Fig. 6d-g, in RP11-241J12.3 overexpressing HCC cells, there was an evident increase in the enhanced γ-H2AX DNA damage foci. Therefore, we hypothesized that, in this system, abnormal overexpression of MSH3 resulted in the preferential formation of Mutsβ by combining with MSH2, causing Mutsα collapse, and leading to further mismatch repair deficiency and tumorigenesis [18].

As previously described, pyruvate accumulation reduced DNA damage. Moreover, upregulation of PC further decreased of pyruvate levels and exacerbated DNA damage to promote HCC aggressiveness. Thus, we verified that RP11-241J12.3 contributed to hepatocellular carcinoma by regulating pyruvate metabolism through targeting PC and disrupted the DNA MMR system by disintegrating the MMR complex.

### RP11-241J12.3 was highly expressed in human HCC

**Fig. 7:**
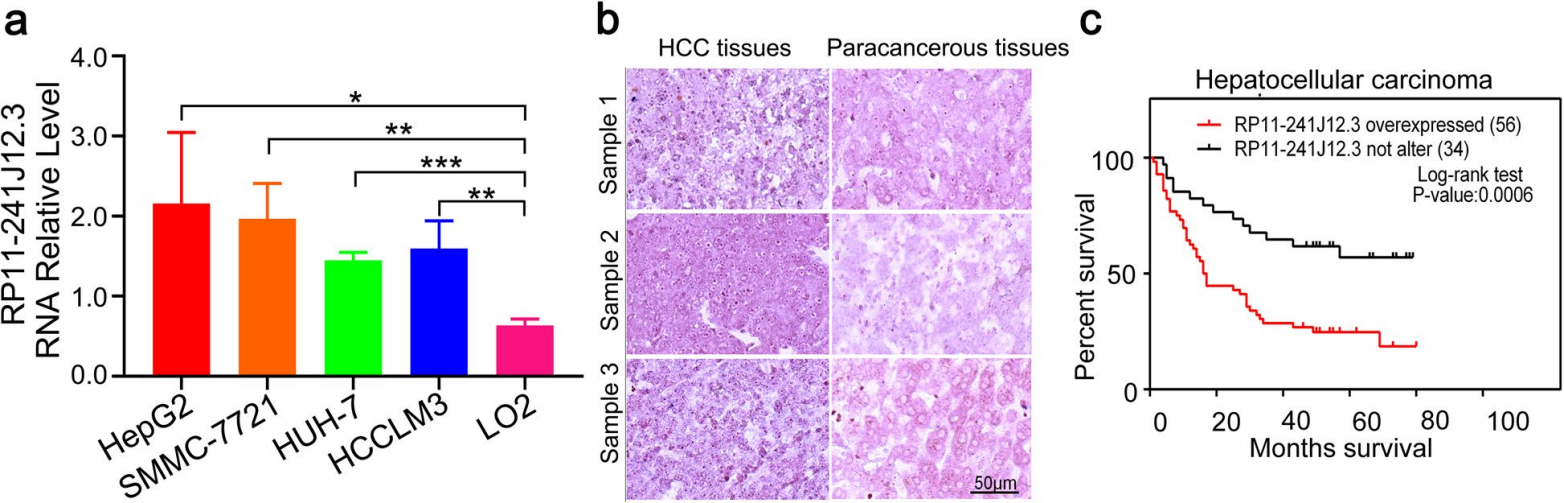
RP11-241J12.3 was highly expressed in human liver tumours and closely related to survival of clinical patients. **a** RP11-241J12.3 expression in HepG2, HCCLM3, HUH7 SMMC-7721 and LO2 cells. qPCR analysis showing that RP11-241J12.3 is more highly expressed in HCC cells than in LO2 cells. Data are normalized to *GAPDH* and represent the mean ± SD of at least three replicate experiments. **b** Representative images of RP11-241J12.3 expression in HCC tissues and paracarcinomatous tissues. 56 out of 90 hepatocarcinoma tissues displayed high expression of RP11-241J12.3 compared with paracarcinomatous tissues. **c** Relationship between survival (months) of patients and RP11-241J12.3 expression. Higher expression of RP11-241J12.3 correlates with shorter survival post-surgery

In summary, we demonstrated that lncRNA RP11-241J12.3 promotes cell proliferation, accelerates cell invasion, regulates pyruvate metabolism and impairs the DNA MMR system. All these findings indicated that RP11-241J12.3 functions as a proto-oncogene. Furthermore, we detected the expression of RP11-241J12.3 in HCC cells (HepG2, HCCLM3, HUH7 and SMMC-7721) and normal hepatic cell line (LO2). Compared with the LO2 cells, qPCR analysis showed that RP11-241J12.3 was more highly expressed in hepatoma cells (Fig. 7a).

To further verify the oncogenic role of RP11-241J12.3, we analyzed the expression of lncRNA RP11-241J12.3 in microarrays of hepatocarcinoma tissues and hepatocarcinoma tissues from 90 clinical samples by in situ hybridization (ISH). The result showed that 56 out of 90 hepatocarcinoma tissues displayed high or very high expression of RP11-241J12.3 compared with paracarcinomatous tissues (Fig. 7b). Moreover, overexpression of lncRNA RP11-241J12.3 was found to be negatively correlated with the length of patient survival after surgery (Fig. 7c). These findings indicate implicate RP11-241J12.3 as a potential diagnostic marker for HCC. We further analyzed the relationship between RP11-241J12.3 expression and other characteristics of patients including age, sex, tumor size, liver cirrhosis, Edmondson grade and clinical stage and found that RP11-241J12.3 was closely related only to tumor size and clinical stage (Table 2). These findings provided further evidence of the potential of RP11-241J12.3 as a diagnostic marker and therapeutic target in HCC.

**Table 2:** Correlation between lncRNA 241J12.3 expression and clinical features of HCC patients (*N* = 80)

Variables		LncRNA RP11-241J12.3 expression		***χ***^**2**^	*P -value
		High (*N* = 48)	Low (*N* = 32)		
Age	>55	21 (26.25%)	14 (17.5%)	1.905	0.066
	≤55	27 (33.75%)	18 (22.5%)		
Sex	Male	45 (56.25%)	27 (33.75%)	0.188	0.171
	Female	3 (3.75%)	5 (6.25%)		
Liver cirrhosis	with	22 (27.5%)	11 (13.75%)	0.611	0.308
	without	26 (32.5%)	21 (26.25%)		
Tumor size (cm)	>5cm	34 (42.5%)	14 (17.5%)	2.347	0.015*
	≤5cm	14 (17.5%)	18 (22.5%)		
Tumor number	Solitary	45 (56.25%)	31 (38.75%)	0.375	0.530
	Multiple	3 (3.75%)	1 (1.25%)		
Edmondson grade	I+II	27 (33.75%)	24 (30%)	0.087	1.059
	III	21 (26.25%)	8 (10%)		
Clinical stage	1–2 stage	24 (30%)	16 (20%)	5.104	0.001**
	≥3 stage	24 (30%)	16 (20%)		

The median expression level was used as the cutoff. Thirty-two patients were classified as low lncRNA 241J12.3 expression and 48 were classified as high expression. Pearson’s chi-square tests were used for analysis of correlations between lncRNA 241J12.3 levels and clinical features

## Discussion

HBx playing a significant role in epigenetic modifications, signaling pathways, cell cycle and apoptosis, and especially DNA damage and metabolic disturbance [4–6, 19, 20]. HBx has been reported to cause DNA damage by interacting with several DNA damage-binding protein to inactivate the early DNA damage response. Furthermore, the C-terminal region is necessary for the production of reactive oxygen species production and 8-oxoguanine, which play crucial roles in mitochondrial DNA damage [21, 22]. HBx expression in HCC cells was shown to result in disruption of the balance of some metabolites, such as glucose and and amino acids [7].

LncRNAs are a significant class of regulator including alternative splicing, epigenetic regulation, and microRNA-like molecules, involved in many biologically complex processes and various cancers [23]. Although some studies have showed that several HBx-associated lncRNAs act as tumor suppressors or oncogenes in HCC, many HBx-related lncRNAs with complicated functions and clinical significance remain to be detected [11, 24, 25]. Through microarray analysis of Ad-HBx– and Ad-N–infected HCC cells, we verified that HBx caused changes in the expression of numerous lncRNAs. Some of the differentially expressed lncRNAs, might also be carried by HCC patients and participate in liver tumorigenesis.

In this study, we identified RP11-241J12.3 as a novel lncRNA and showed that its expression was greatly downregulated by HBx. Despite our expectation that this lncRNA might play a role as a suppressor gene, RP11-241J12.3 was found to play a significant role in HCC aggressiveness as an oncogene that promoted proliferation and migration of HCC cells *in vitro* and *in vivo*. Our previous study indicated that HBx inhibited DNA synthesis and induced G2/M arrest in the early stages of the function of HBx [13]. We confirmed that RP11-241J12.3 overexpression upregulated the expression of PC and MSH3, which are involved in pyruvate metabolism and the DNA MMR system, respectively, thus providing further evidence of its role in glycometabolism and DNA replication [26]. As the target protein of RP11-241J12.3, PC maintains the balance of pyruvate metabolism and is closely related to the DNA MMR system. Therefore, we speculated that HBx possibly affected pyruvate metabolism in glycometabolism and the DNA MMR system by regulating the expression of RP11-241J12.3. Furthermore, the ISH results indicated that after DNA synthesis is inhibited in the early response after HBV infection, HBx downregulates RP11-241J12.3 to inhibit the DNA MMR system, causing DNA damage. However, in contrast, HCC cells exhibit abnormal proliferation along with accelerated DNA synthesis and an increase in the DNA mismatch rate, indicating that RP11-241J12.3 expression is upregulated. Thus, the observation that RP11-241J12.3 is downregulated by HBx in addition to its oncogenic features were not contradictory. We also speculated that RP11-241J12.3 is related to HBx expression in that the function and location of HBx change depending on its expression [27]. Therefore, in the next step, we plan to investigate the connection between the expression of HBx and RP11-241J12.3.

In recent years, a handful of lncRNAs have been found to be closely connected with metabolism and DNA damage [28, 29]. LncRNA NBR2 was induced by the LKB1-AMPK pathway under energy stress and lncRNA-JADE was induced after DNA damage [24, 30]. In this study, lncRNA RP11-241J12.3 was downregulated by HBx and was confirmed to participate in pyruvate metabolism by targeting PC, in addition to regulating MSH3 in the DNA MMR system. Further analysis indicated that PC and MSH3 are regulated by RP11-241J12.3, which is expressed at high levels in HCC tissue. Some reports have confirmed that PC expression is greatly enhanced in cancerous tissues compared with noncancerous tissues [31, 32]. Although some clinical reports have shown that loss of proteins related to the DNA MMR system caused tumorigenesis in cervical cancer [33, 34], it was noted that downregulation of MMR-related proteins is not associated with HCC occurrence [35]. Others demonstrated that MSH2, which interacts with MSH3, was expressed at high levels in colorectal cancers in a manner that was related the severity of the grade of malignancy and lower rates of survival [36, 37]. These findings are in accordance with our expectations for MSH3 expression in HCC. In future studies, we will explore the expression of MSH3 in HCC, and its relationship with RP11-241J12.3.

In summary, we identified the novel lncRNA RP11-241J12.3 and demonstrated that it acts as an oncogene in HCC cells by regulating PC or MSH3 proteins. These findings further clarify the mechanism underlying the effects of HBx on pyruvate metabolism and DNA damage, and implicate RP11-241J12.3 as a potential target for interference in pyruvate embolism and the occurrence of HCC. Our study also provide evidence that supports the potential of lncRNAs as epigenetic therapies, especially for the treatment of HBV-related HCC. Additionally, we reveal that RP11-241J12.3 interacts with PC and is located in the cytoplasm close to the nucleus, with high expression in HCC tissues compared with paracarcinomatous tissues. The survival analysis indicated that RP11-241J12.3 is related to the survival of HCC patients, with high expression related to a sharply reduction in overall tumor-free and postoperative survival. In general, lncRNA RP11-241J12.3 is implicated as a prognostic and diagnostic biomarker in HCC.

## Materials and methods

### Cell culture

HepG2, HCCLM3 and SMMC-7721 human HCC lines were purchased from the American Type Culture Collection and cultured in Dulbecco’s modified Eagle medium (DMEM; Gibco) supplemented with 10% fetal bovine serum (FBS; Gibco) at 37°C in a humidified atmosphere under 5% CO_2_.

### Lentiviral vectors, plasmid vectors and recombinant adenovirus

Lentivirus (Ubi-MCS-SV40-EGFP-IRES-puromycin, Shanghai GeneChem, China) overexpressing RP11-241J12.3 (Lv-241J12.3) and control (Lv-control). Short hairpin RNAs (shRNAs) were specifically designed to knockdown lncRNA RP11-241J12.3. Two shRNAs targeting LncRNA241J12.3-4 and negative control shRNA were designed and cloned into plasmid vectors (pGPU6/GFP/Neo or pGPU6/Neo, GenePharma, Shanghai, China). These shRNAs were designated sh-241J12.3-4, sh-241J12.3-120, and sh-NC, respectively. The infection and transfection efficiencies were confirmed by qRT-PCR**.** Recombinant adenovirus (Ad-HBx) expressing HBx was prepared as described previously [13]. Empty Ad-N served as a control adenovirus. The shRNA sequences are shown in the supplementary data (Information S1).

### Transfection, infection and construction of stable cell lines

HepG2, HCCLM3 and SMMC-7721 cells (3×10^5^) were seeded in a six-well plate. After cell adherence, plasmid expressing shRNA against lncRNA RP11-241J12.3 (2.5 μg) was transfected using X-tremeGENE HP DNA Transfection Reagent (Roche) for HepG2 or HCCLM3 cells and DNA-In Transfection Reagent (MTI-Globalstem) for SMMC-7721; untreated or scramble-control shRNA (sh-NC) transfected cells were used as controls. Similarly, cells were transduced with lentivirus (Lv-NC and Lv-241J12.3) at a multiplicity of infection (MOI) of 10 (HepG2 or SMMC-7721) or 20 (HCCLM3) with polybrene (5 μg/ml). For construction of stable cell lines, at 48 h after transduction with lentivirus, cells were screened with puromycin (10 μg/ml) to screen and maintained in culture in the presence of 6 μg/ml puromycin. SMMC-7721 cells were transduced with the Lv-241J12.3 and Lv-NC to obtain HCC cells stably overexpressing RP11-241J12.3 and its control, respectively.

### RNA isolation, quantitative real time PCR (qRT–PCR) and lncRNA microarray

Total RNA was isolated using TRIzol (Invitrogen) reagent and was reverse transcribed with the PrimeScript™ RT reagent Kit (TaKaRa). Then, the cDNA was applied for qPCR using the SYBR Green (TaKaRa) with gene-specific primers. Real-time PCR was performed on a CFX96 Touch Real-time PCR detection system (Bio-Rad). The data were normalized to *GAPDH or β-actin*.

For lncRNA microarray analysis, total RNA was isolated from Ad-HBx– or Ad-N–infected HepG2 cells, reverse transcribed to cDNA, labeled with Cy3-labelled-CTP. After hybridization, the samples were were scanned with anMicroarray Scanner (Agilent). The specific qRT-PCR primers were shown in Table 1.

### 5-Ethynyl-2'-dexyuridine (EdU) incorporation assay

We utilized EdU incorporation assays to investigate DNA synthesis after lncRNA RP11-241J12.3 knockdown. HepG2 and HCCLM3 cells transfected with Sh-241J12.3-4 or Sh-241J12.3-120, or Sh-NC, or untreated were seeded in 96-well plates and cultured in DMEM supplemented with 10% FBS for 70 h before labelling using 50 μM EdU. Then cells were fixed by 4% paraformaldehyde and stained with Apollo®488 fluorescent dye and Hoechst33342. The proliferating cells were imaged by fluorescent microscopy (Olyumpus).

### Monolayer colony-formation assay and cell proliferation assay

For monolayer colony-formation assay, SMMC-7721 cells stably expressing or silenced expression of the objective gene (500) were seeded in six-well plates in triplicate, and cultured in medium supplemented 10% FBS under appropriate conditions for 2–3 weeks. Subsequently, colonies were fixed with 4% formaldehyde and stained with 5% crystal violet dye.

In cell proliferation assay, 1,000 SMMC-7721 cells (stably expressing the objective gene) or HCCLM3 cells (objective gene knockdown) were seeded in 96-well plates and quantified using the WST-8 reagent (Dojindo Laboratories, Kumamoto, Japan) at the specified time according to the manufacturer’s instructions.

### Transwell invasion assay

Transwell membranes (pore size 8μm, Millipore) were coated with 100 μl Matrigel (Corning; Matrigel:serum-free medium, 1:10). SMMC-7721 cells (8×10^4^) in 200 μl serum-free medium were seeded into the upper chamber, while 900 μl medium containg with 10% FBS was added to the lower chamber. The cells were cultured for 24 h. Then, the migrated cells adhering to the lower surface were fixed with 4% paraformaldehyde and stained with 0.5% crystal violet dye. The number of cells were counted in 10 randomly selected visual fields viewed under a microscope.

### BALB/c xenografted tumour growth *in vivo*

All experimental procedures were approved by the Animal Care and Use Committee of Sichuan University. Balb/c nude mice (female, 6–8 weeks) were purchased from the Laboratory Animal Center of Sichuan University. For xenograft models, single cell suspensions were obtained by trypsinization, washed twice and resuspended with serum-free DMEM medium. SMMC-7721 cells, stably infected with Lv-241J12.3 or Lv-NC (5×10^6^/100 μl) were injected subcutaneously into the left and right rear flanks of each mouse respectively. Tumor size was measured every week, and mice were monitored routinely for 6 weeks. Then, the mice were sacrificed for histology analysis.

### Immunohistochemistry and hematoxylin-eosin (HE) staining

For the *in vivo* cell proliferation experiments, immunohistochemistry was performed on paraffin-embedded tissue sections using an anti-Ki-67 antibody (Abcam). Briefly, after complete dewaxing and antigen-retrieval, tissues were incubated for 15 min at room temperature with goat serum and overnight at 4°C with anti-Ki-67 antibody (1:1000). Then, the sections were incubated with horseradish peroxidase-conjugated rabbit anti-goat antibody (1:10000), visualized with 3, 3′-diaminobenzidine (DAB) and stained with hematoxylin. The immunohistochemical staining images were captured by the digital camera (Carl Zeiss). The percentage of Ki-67^+^ cells in the digital photomicrographs were assessed using an automated software analysis program (Carl Zeiss). For HE staining, tissues were embedded with paraffin and section (thickness 4–6 mm) were prepared for HE staining. Sections were evaluated under a bright-field microscope.

### RNA pull-down assay and RNA binding protein immunoprecipitation (RIP)

Biotin-labeled RNA was transcribed with T7/SP6 RNA polymerase (Promega) and the Biotin RNA Labeling Mix (Roche), and purified with RNeasy Mini Kits (QIAGEN) *in vitro*. The correct secondary structure of the biotinylated RNA (3 μg) was formed in RNA structure buffer as previously described [38]. The RNA was then mixed with HepG2 cell cytoplasm extract (approximately 1 mg protein) in 500 μl immunoprecipitation (IP) buffer (Thermo) and incubated for 1 h at room temperature. Then, 50 μl prepared streptavidin agarose beads (Invitrogen) were added to each reaction and incubated for a further 1 h at room temperature. Beads were briefly washed six times with RIP buffer and boiled in sodium dodecyl sulfate (SDS) buffer. The retrieved samples were resolved by sodium dodecylsulphate polyacrylamide gel electrophoresis (SDS-PAGE), and identified using silver staining and mass spectrometry (MS) analysis. RIP was performed with human PC-specific antibodies (Abcam, ab110314) using the Magna RIP™ RNA-Binding Protein Immunoprecipitation Kit (Millipore) according to the manufacturer’s instructions. HepG2 cells were lysed with cell lysis buffer containing with RNase inhibitors and protease inhibitor cocktail. RNA was detected by qPCR.

### Western blotting

Western blotting was performed standardly as previously described [39]. Briefly, Tumor tissues were homogenized in RIPA buffer containing protease inhibitor cocktail and phenylmethanesulfonyl fluoride (PMSF). SMMC-7721 and HepG2 cells stably infected with Lv-241J12.3 or Lv-NC. Cells were lysed in RIPA buffer containing protease inhibitor cocktail and PMSF. Total proteins were separated by SDS-PAGE and transferred to polyvinylidene fluoride (PVDF) membranes, which were incubated overnight at 4°C with primary detection antibodies against human Ki67 (Abcam), MSH3 (Labome, sc-271079) and β-actin (Abcam). The membranes were then incubated for 1 h at 37°C with horseradish peroxidase-conjugated secondary detection antibodies. Protein bands were detected using an enhanced chemiluminescence system (Millipore) and iBright™ CL1000 Imager (Thermo). Quantitative densitometric analysis was performed using iBright™ software.

### Intracellular metabolites assay

The PC activity and level of oxaloacetate (OAA) in HCC cells were detected using PC Activity Colorimetric Assay Kit (BioVision) and OAA Fluorometric Assay Kit (BioVision), respectively, as per the manufacturer’s instructions.

### Immunofluorescence analysis

HepG2 and HCCLM3 cells were fixed with 4% paraformaldehyde for 20 min and permeabilized with 0.5%Triton X-100 for 15 min. To detect the PC location, the fixed cells were incubated with anti-PC antibody (Abcam, 1:200) overnight at 4°C. The cells were then incubated with TRITC-conjugated goat anti-mouse IgG (Abcam, 1:1000) for 1 h at 37°C, and counterstained with DAPI at room temperature for 5 min. The images were acquired by a fluorescence microscope.

### LncRNA fluorescence *in situ* hybridization (FISH)

For detection of the lnc-RP11-241J12.3 RNA, HepG2 cells were seeded onto glass coverslips and cultured at 37°C for 12 h. Then, the cells were fixed in 4% formaldehyde (pH 7.4) containing 10% acetic acid for 15 min. The cells were then permeabilized in PBS containing 5 mM vanadyl ribonucleoside complex (New England BioLabs) and 0.5% Triton X-100 on ice for 5 min, and then rinsed in PBS three times (10 min per wash) and once with 2× SSC buffer. The RNA probe (Exiqon) was labelled with double-DIG in a hybridization instrument (UVP) at 55°C for 2 h. Hybridized probes were detected by incubation with FITC-labelled anti-DIG antibody (Roche) at 37°C for 2 h. For RNA-FISH co-localization, cells were observed using a Zeiss LSM 800 laser scanning confocal microscope. The specific sequence of the RP11-241J12.3 detection probe are shown in the supplementary data (Information S2).

### LncRNA *in situ* hybridization

The HCC tissue microarray (no. HLiv-HCC180Sur-04; Shanghai Outdo Biotech Company) comprised 90 liver tumor samples and 90 control liver tissue samples. The double-DIG-labeled lncRNA-RP11-241J12.3 probe was purchased from Exiqon. Briefly, the tissue microarray was dewaxed in xylene, rehydrated by immersion in an ethanol dilution series and digested with proteinase K (20 μg/ml) for 15 min at 37°C. Hybridization of tissue sections was performed by incubation with the appropriate probe for 2 h with 55°C, and detected using the anti-DIG antibody conjugated alkaline phosphatase (Roche) with NBT-BCIP (Roche) as the substrate.

### Statistical analysis

Data were presented as the mean ± standard deviation (SD) of three independent experiments. Student’s *t*-test was used for statistical comparison between groups. One-way ANOVA followed by Tukey’s test was used for multiple comparisons. Pearson’s chi-square tests were used for analysis of correlations between lncRNA levels and clinical features. *P < 0.05, **P < 0.01, ***P < 0.001, ****P < 0.0001. *P-*values <0.05 were considered to indicate statistical significance.

## Supplementary Information

**Additional file 1: Figure S1.** The overexpression and shRNA-mediated knockdown of RP11-241J12.3 in HCC cells. **Table S1.** All nucleotide sequences and annealing temperatures of primers used in qRT-PCR. Table S1 (related to Figs. 1 and 6). **Information S1. **ShRNA sequence for targeting lncRNA 241J12.3 in vetor pGPU6 /Neo and pGPU6 /GFP /Neo. **Information S2.** The sequence of probe for lncRNA RP11-241J12.3 in FISH and ISH.

## Data Availability

The data is available with the corresponding author and will be provided upon the legitimate request.
