# Supplementary material for: Long noncoding RNA RP11-241J12.3 targeting pyruvate carboxylase promotes hepatocellular carcinoma aggressiveness by disrupting pyruvate metabolism and the DNA mismatch repair system
Source: Mol Biomed. 2022 Feb 5;3:4. doi: 10.1186/s43556-021-00065-w (PMC8816999; doi:10.1186/s43556-021-00065-w)
Supplement: Supplementary file 1 — Additional file 1: Figure S1. The overexpression and shRNA-mediated knockdown of RP11-241J12.3 in HCC cells. Table S1. All nucleotide sequences and annealing temperatures of primers used in qRT-PCR. Table S1 (related to Figs. 1 and 6). Information S1. ShRNA sequence for targeting lncRNA 241J12.3 in vetor pGPU6 /Neo and pGPU6 /GFP /Neo. Information S2. The sequence of probe for lncRNA RP11-241J12.3 in FISH and ISH. [file 43556_2021_65_MOESM1_ESM.docx]

**Long noncoding RNA RP11-241J12.3 targeting pyruvate carboxylase promotes** **hepatocellular carcinoma aggressiveness by disrupting** **pyruvate metabolism and the DNA mismatch repair system**

Liuliu Cheng^1^†, Shichuan Hu^1^†, Jinhu Ma^1^†, Yongheng Shu^1^, Yanwei Chen^1^, Bin Zhang^1^, Zhongbing Qi^1^, Yunmeng Wang^1^, Yan Zhang^2^, Yuwei Zhang^3^, Ping Cheng^1^*

**Affiliations**

^1^State Key Laboratory of Biotherapy and Cancer Center/Collaborative Innovation Center for Biotherapy, West China Hospital, Sichuan University, Chengdu 610041, Sichuan, PR China.

^2^Department of Thoracic Oncology, Cancer Center and State Key Laboratory of Biotherapy, West China Hospital, Sichuan University, Chengdu 610041, Sichuan, PR China.

^3^Division of Endocrinology and Metabolism, State Key Laboratory of Biotherapy, West China Hospital, Sichuan University, Chengdu 610041, Sichuan, PR China.

†These authors contributed equally to this work.

*Corresponding author: Prof. Ping Cheng, State Key Laboratory of Biotherapy and Cancer Center, West China Hospital, Sichuan University, and Collaborative Innovation Center for Biotherapy, 17 People's South Road, Chengdu 610041, China. Email: ping.cheng@foxmail.com, Tel: 86-28-85164060, Fax: 86-28-85164060.

**
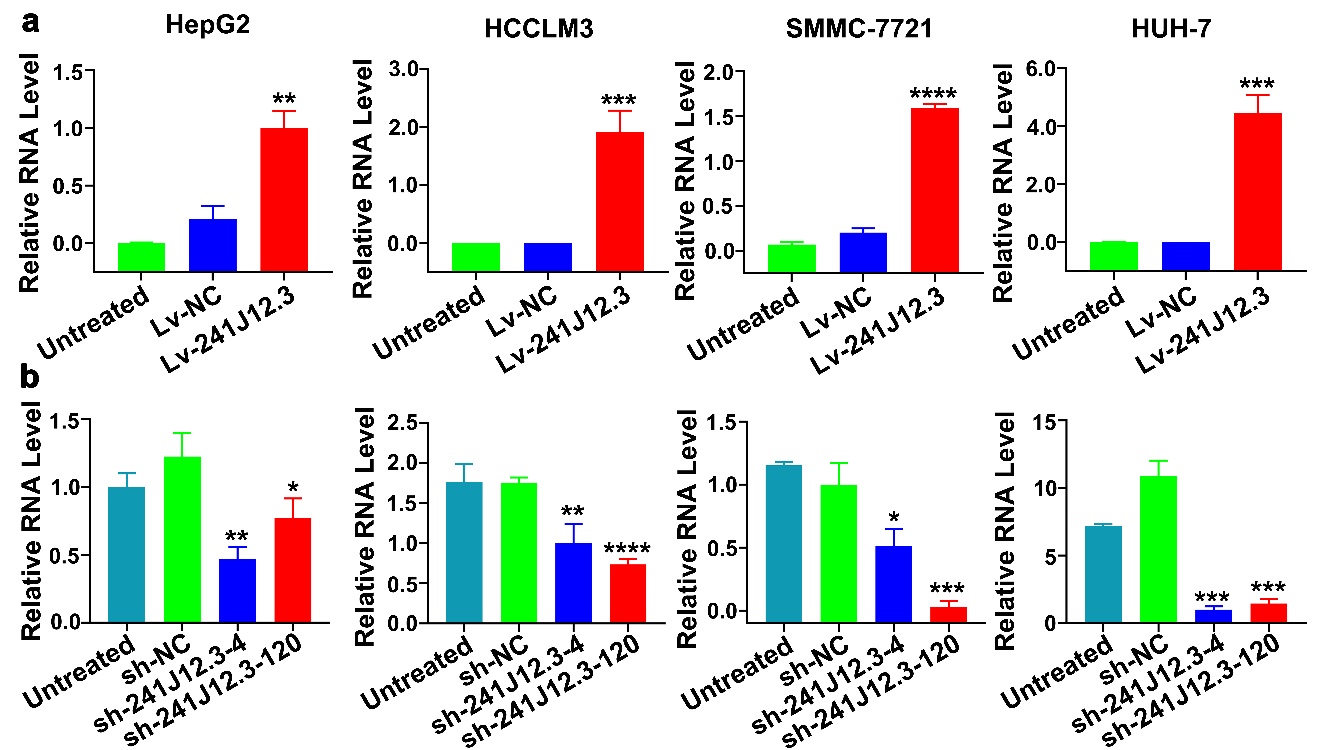
**

**Figure S1. The** **overexpression and shRNA-mediated knockdown of RP11-241J12.3 in HCC cells. (a)** HepG2, HCCLM3, SMMC-7721 and HUH7 cells were transduced with lentivirus overexpressing lncRNA RP11-241J12.3 and screened by qRT-PCR. **(b)** ShRNAs (sh-241J12.3-4 or 120, pGPU6/GFP/Neo) against lncRNA RP11-241J12.3 were transfected into HepG2, HCCLM3, SMMC-7721 and HUH7 cells to decrease the expression of the objective lncRNA compared with sh-NC cells was confirmed by qRT-PCR.

**Table s1. All nucleotide sequences and annealing temperatures of primers used in qRT-PCR.** **Table s1 (related to Figure1 and Figure 6)**

| Gene | Primer nucleotide sequences | Annealing temperature |
| --- | --- | --- |
| GAPDH | F:5'-GGGAAACTGTGGCGTGAT-3'  R:5'-GAGTGGGTGTCGCTGTTGA-3' | 60°C |
| β-ACTIN | F:5'-ACCCTGAAGTACCCCATCGAG-3'  R:5'- ACATGATCTGGGTCATCTTCTCG-3' | 60°C |
| ENST00000511272 | F:5'- GTCAGTTGTTCCTCCAGTTCCG-3'  R:5'-TCAGCTCCCAGGTCACCCA-3' | 60°C |
| ENST00000506122 | F 5'-GATCCCTCCTTAGGACCAACAA-3'  R 5'-GGCATACCTGGGTTTCCTCTT-3' | 60°C |
| ENST00000430583 | F:5' –AAGGGAAGATTCTCAAAGACCAG-3'  R:5'-GTCATCAAAGGTTACAAAGGCAA-3' | 60°C |
| ENST00000477387 | F:5'-AGAGCTTCTTTACCTGATTTGCTG-3'  R:5'-CTAATCCTTCCTCCTTTGCGTC-3' | 60°C |
| ENST00000511272 | F:5'-GTCAGTTGTTCCTCCAGTTCCG-3'  R:5'-TCAGCTCCCAGGTCACCCA-3' | 60°C |
| ENST00000457635 | F 5'-AAATCAGCAGCAGCAGTCCTT-3'  R 5'-CAGCTTCCAATTCTTTGTCCC-3' | 60°C |
| ENST00000507583 | F:5'-CCATGATCCAAAGGAACCAGA-3'  R:5'-GTAGTTTCAAAACTCAGACCACCAA-3' | 60°C |
| HIT000320855 | F:5'-GGGAAACTGTGGCGTGAT-3'  R:5'-GAGTGGGTGTCGCTGTTGA-3' | 60°C |
| ENST0000050460 | F:5'-GGGTCATTTCCACATGCTTTAT-3'  R:5'-TCCTAGTCTGTCCTGCGTCCT-3' | 60°C |
| ENST00000333487 | F:5'-CGGAGGCTTGACAAGGTTG-3'  R:5'-GAGGTGCTTGAAGGAGTGGG-3' | 60°C |
| MSH2 | F: 5'-GGACTGCATCTTAGCCCGAGTAGG-3'  R: 5'-GCCCATGCTAACCCAAATCCATCG-3’ | 60°C |
| MSH3 | F :5'-GCCTTGTCCGAGCAAACAGAGG-3'  R :5'-AACTGCCTGGAAAGCATGGCTG-3' | 60°C |
| MLH1 | F :5'-TTATCCAGCGGCCAGCTAATG-3'  R :5'-GCCTCCCTCTTTAACAATCACTT-3' | 60°C |
| PC | F : 5'-TGAAGTCTGGCAACTCGGAC-3'  R :5'-TGATGAGATCGCCCAGCATC-3' | 60°C |

**Information S1: ShRNA sequence for targeting lncRNA 241J12.3 in vetor pGPU6 /Neo and pGPU6 /GFP /Neo.**

(pGPU6/Neo)Sh-241J12.3-4: GCCGTCAATTAAGATGCTGAG

(pGPU6/Neo)Sh-241J12.3-120: ACCCAGCTCCTCTACCAAAGT

(pGPU6/Neo)ShRNA-NC: GTTCTCCGAACGTGTCACGT

(pGPU6/GFP/Neo)Sh-241J12.3-4: GCCGTCAATTAAGATGCTGAG

(pGPU6/GFP/Neo)Sh-241J12.3-120: ACCCAGCTCCTCTACCAAAGT

(pGPU6/GFP/Neo)ShRNA-NC: GTTCTCCGAACGTGTCACGT

**Information S2: The sequence of probe for lncRNA RP11-241J12.3 in FISH and ISH .**

The sequence of probe : /5DigN/CGCTGGTTCGCTTGGCATA/3DigN/
